# Provenance, diagenesis, and depositional environment of Miocene Kamlial Formation, Azad Jammu and Kashmir, Sub Himalayas, Pakistan: Evidences from field observations and petrography

**DOI:** 10.1016/j.heliyon.2024.e24309

**Published:** 2024-01-06

**Authors:** Musa Khan, Rehan Khan, Shams Ul Islam, Asad Khan, Yanmei Zhong, Fuad A. Awwad, Emad A.A. Ismail

**Affiliations:** a Key Laboratory of Metallogenic Prediction of Nonferrous Metals and Geological Environment Monitoring, Ministry of Education, School of Geosciences and Info-Physics, Central South University, Changsha 410083, China; b Institute of Geology, University of Azad Jammu and Kashmir, Muzaffarabad, Azad Jammu and Kashmir, Pakistan; c Department of Geology, FATA University, FR Kohat 26100, KP, Pakistan; d School of Resource and Environmental Sciences, Wuhan University, Wuhan 430079, China; e Department of Quantitative analysis, College of Business Administration, King Saud University, P.O. Box 71115, Riyadh 11587, Saudi Arabia

**Keywords:** Kamlial formation, Petrography, Field observations, Depositional environment, Diagenesis

## Abstract

Petrographical characterization and field observations were caried out to evaluate Kamlial Formation in Bagh district, Azad Jammu and Kashmir. Based on detailed petrography, the lithic arenite consisted of quartz (20–25%), feldspar (7–11%), rock fragments (20–37%), cementing materials (11–21%), and accessory minerals. Grains are mostly angular to subrounded and poorly to moderately sorted. The analysis revealed that the lithic arenite is mineralogically immature; also, the current activity during the time of deposition was low. Polycrystalline quartz indicates that the sandstone was derived from metamorphic source, while monocrystalline quartz indicates a granitic origin. Quartz having an angular shape suggests the source rock was near the depositional site, while quartz having a rounded shape represents long transportation. The presence of feldspar in the lithic arenite suggests the rocks were deposited at high relief or cold temperatures. Primary porosity in sandstone was reduced by calcite cements around the grain, while secondary porosity was developed by fracturing of quartz and feldspar. Tectonic uplift in the study area was demonstrated by fractured quartz and mica in thin sections. Field observations of various sedimentary structures were observed such as load casts, ripple marks, and mud cracks, etc. The presence of conglomerates and load casts in the study area indicates that the Kamlial sandstone was deposited by fluvial and shallow marine environment. Furthermore, the ripple marks indicate that the tidal flat environment controlled the deposition of the sediments.

## 1 Introduction

The Himalayan Foreland Basin is one of the largest and most dynamic terrestrial basins in terms of area and dynamic processes. There are several sub-basins in this area, separated by several Pre-Neogene basements [1]. The Indus and the Baluchistan basins have been identified as the two major sedimentary basins in Pakistan. During the Western Himalayan orogeny, the Kamlial Formation preserved records of tectonic processes, climatic conditions, sedimentation patterns, and drainage organization [2].

**Fig. 1:**
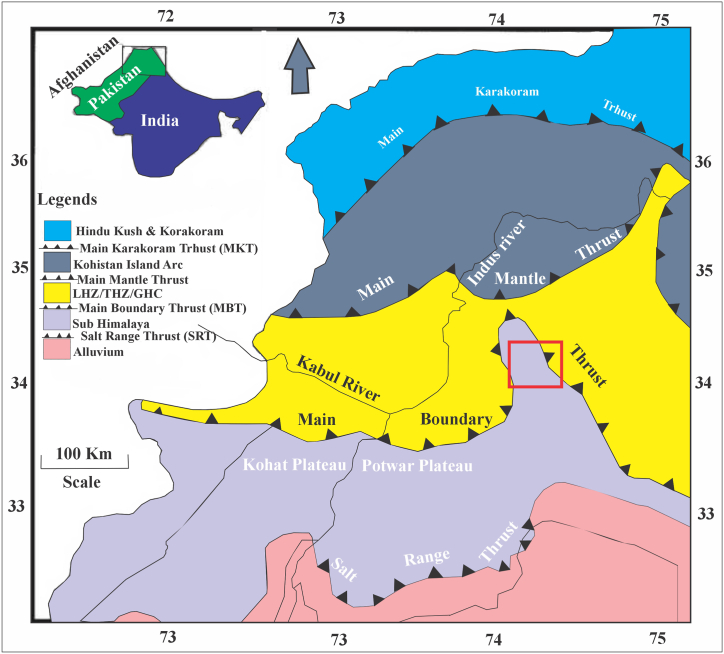
Regional geological map of the study area. From north to south this map includes; Hindu Kush and Karakoram, Kohistan Island Arc, Himalayas. Red rectangle showing the location of the area. LHZ = Lesse Himalayan Zone, THZ = Tethys Himalayan Zone, GHC = Greater Himalayan Complex. Map modified after [6]. (For interpretation of the references to color in this figure legend, the reader is referred to the Web version of this article.)

During the Tertiary Himalayan collisions, sedimentary rocks in the Dhirkot area of Azad Jammu and Kashmir, Sub-Himalayas, Pakistan, have been deformed (Fig. 1). In this area, folds, faults, joints, and fractures are observed. Major structures include the Dhirkot fault and the Chamiati syncline. There were fractures along the Dhirkot Fault during the Kashmir earthquake on October 8th, 2005 [3]. Chamiati syncline contains two rock units, the Murree Formation, and the Kamlial Formation. The Murree Formation consists of fine-to medium-grained grey sandstone, clays, and conglomerates. Kamlial Formation, consists of blue-grey sandstones, khaki sandstones, grey sandstones, reddish sandstones, reddish-brown clays, and khaki-brown clays. These rocks were formed during the Himalayan orogeny [4,5].

The town of Dhirkot is a popular tourist destination located 132 km from Islamabad, the capital city. It is in Azad Jammu and Kashmir's district Bagh. This area is characterized by high relief topography and a mountainous landscape. The altitude ranges from 1676 m to 2000 m. Summer temperatures range from 25 to 35 °C, and winter temperatures are from −5 to 5 °C. Annual rainfall averages between 2400 and 2850 mm. This area has 100% relative humidity. There is a tropical climate as well as a temperate climate in this area. It is extremely cold in the winter and pleasant in the summer [7].

The surrounding area has been explored by geoscientists in the past. According to Ref. [8], there are considerable mineral and geological resources in the southern Hazara area as well as adjacent areas of Azad Jammu and Kashmir. Tectonic maps of Pakistan were prepared in Ref. [9]. The Murree Formation in Pakistan has been studied by Ref. [10]. Tectonics, metamorphism, and stratigraphy of Hazara Kashmir Syntaxis were examined by Refs. [11,12]. The Kamlial Formation of the Kohat Basin was studied in detail by Ref. [13]. An investigation of Nauseri granite in the Neelum Valley of Azad Jammu and Kashmir was conducted by Ref. [14]. The geology and tectonics of Pakistan were described by Ref. [15]. [6] has conducted several studies on the petrography of the Kamlial and Chinji Formations, located on the southwest Kohat Plateau in northwest Pakistan. According to Ref. [16], the biostratigraphy of the upper Cretaceous of Hazara and Paleogene of Azad Jammu and Kashmir, NW Himalayas, Pakistan, was discussed. The stratigraphy of Pakistan has been studied by Ref. [17]. During the 2005 Kashmir earthquake, researchers [18] investigated the behavior of mass movements caused by the earthquake. A study conducted by Ref. [19] investigated the petrography, provenance, and depositional environment of Murree sandstone in Jhelum Valley, district Hattian, and Azad Jammu and Kashmir, Pakistan. [20] has examined the petrography and geochemistry of the Kamlial Formation in the southern Kohat Plateau, Pakistan.

The Kamlial Formation, lies in the Bagh district of Azad Jammu and Kashmir, has received less attention in the past. Based on field observations, petrographic analysis, and litho-stratigraphic study, the provenance, diagenesis, and depositional environment of this formation were evaluated. Therefore, the main objectives of this study were (1) To prepare a geological map of the projected area at a scale of 1:50,000. (2) An area map was prepared illustrating a sample location as well as structures and contours of the studied area. (3) To prepare a lithological log based on the stratigraphy of Muzaffarabad and Dhirkot areas. (4) To determine petrographical observations of the selected rock samples to established model minerology and textural attributes etc. (5) To determine the provenance, diagenesis, and depositional environment of the Kamlial Formation by combining field observations and laboratory data.

## 2 Geological setting

Indian plates moved away between the Middle-Late Jurassic and Early Cretaceous. Due to the northward movement of the Indian plate, the Tethys Ocean was closed and the Himalaya was formed by the collision between the Indian and Eurasian plates [[21], [22], [23], [24], [25], [26], [27]]. According to Refs. [28,29] in the north, the Himalayan terrain ends at the Indus Tsangpu Suture Zone. The Main Mantle Trust (MMT) separates the Kohistan Island Arc from the Indian Plate in the NW Himalayas. The Kohistan Island Arc and the Eurasian Plate are separated by the Main Korakoram Thrust (MKT) [[30], [31]]. Kohistan Island Arc contains highly dense rocks while the Indian and Eurasian Plates contain less dense rocks [[32], [33]]. Rocks in the study area range from Precambrian to Recent, which are found in Muzaffarabad and Dhirkot (Table 1). The stratigraphy of the study area has been modified in accordance with [17,34,35].

**Table 1:** Generalized stratigraphy of study area modified after [17,34,35].

Formation	Age	Lithology
Quaternary alluvium	Recent	Consists of silt, gravel and unconsolidated deposits of clay.
Unconformity		
Dhok Pathan Formation	Late Miocene	Dominantly consists of sandstone, siltstone and clays. Sandstone is grey, fine to medium grained and medium to thick bedded.
Nagri Formation	Late Miocene	Dominantly consists of greenish grey sandstone, siltstone and mudstone. Sandstone is massive and medium to coarse grained. Sandstone and clay ratio is 60-40 %.
Kamlial Formation	Early to Middle Miocene	Mainly sandstone, clays and intraformational conglomerates.
Murree Formation	Early Miocene	Mostly clays, shale and sandstone. Sandstone is red to purple red and fine to medium grained.
Unconformity		
Kuldana Formation	Middle to Late Eocene	Variegated shale with subordinate sandstone. Shale are arenaceous.
Chorgali Formation	Early Eocene	Mostly calcareous shale, limestone and dolomitic limestone.
Margalla Hill Limestone	Early Eocene	Main nodular fossiliferous limestone with shale.
Patala Formation	Late Paleocene	Mainly shale interbedded with marl and limestone.
Lockhart Formation	Early Paleocene	Grey to dark grey limestone with subordinate shale.
Hangu Formation	Early Paleocene	Mainly laterite, bauxite and fireclay.
Unconformity		
Panjal Formation	Permian	Volcanic rocks, dykes and sills.
Unconformity		
Abottabad Formation.	Cambrian	Mainly dolomitic limestone with cherty dolomiteand chert bands.
Unconformity		
Tanol Formation	Precambrian	Schists and Phyllite.
Salkhala Formation	Late Precambrian	Slates.
Hazara Formation	Late Precambrian	Slate, phyllite and shale.

**Fig. 2:**
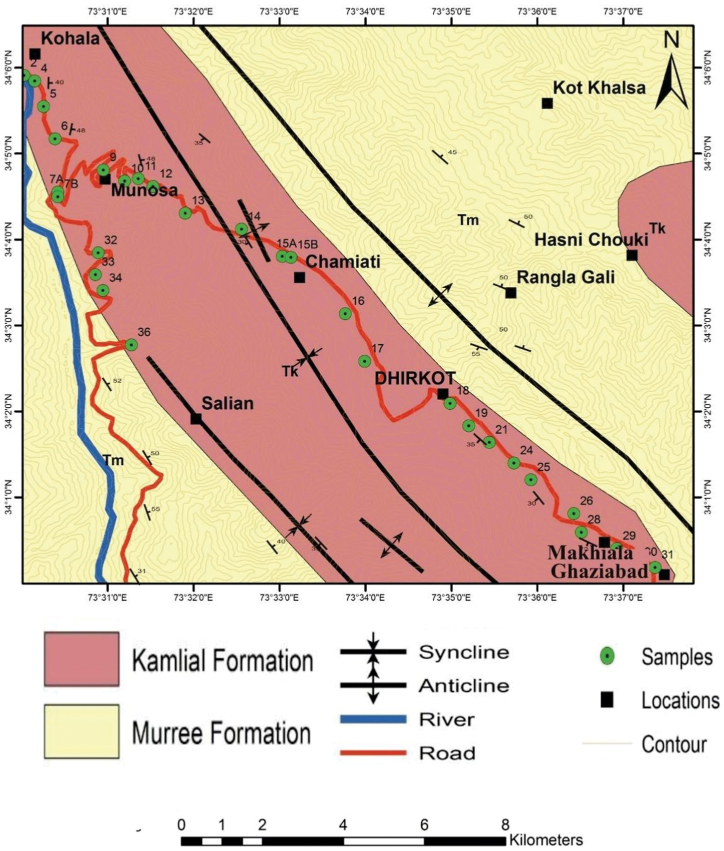
Geological map of the study area showing the Kamlial and Murre Formation.

Research is being conducted in the Hazara Kashmir Syntaxis (HKS) of the Sub-Himalayas (Fig. 1). The Hazara Kashmir Syntaxis is a complex tectonic domain that includes rocks from the Lesser and Sub Himalayas [10]. Several thrust faults are present around the HKS top, including the MBT and Panjal Thrusts (Fig. 1). The HKS is composed of sedimentary, volcanic, and metamorphic rocks of the Precambrian to Neogene [[12], [36]]. The rocks under investigation are Miocene clastic sediments of the Kamlial Formation (Fig. 2). There are three sections of the field, namely Kohala to Dhirkot, Dhirkot to Ghaziabad, and Khapaddar to Kohala. Stratigraphic Committee of Pakistan renamed the "Kamlial beds" of [37] as the Kamlial Formation. Kamlial Formation in the study area consists of medium-to coarse-grained greenish grey and bluish sandstones, purple colored shale, and yellow and purple intraformational conglomerates. It differs from the underlying Murree Formation due to its spheroidal weathering and heavy mineral content. Tourmaline dominates epidote in mineral content. Conglomerates and clay are subordinate to the sandstone in the Formation. Sandstone is greenish grey in color, medium-to coarse-grained, cross-bedded, and massive in nature. There are instances where the sandstone is bluish grey, dull red with a salt and pepper pattern, calcareous, and moderately to poorly cemented. [17] indicates that clay can be sandy or silty, chocolate brown or reddish grey, and pale orange in color depending on the section.

## 3 Stratigraphy of the area

**Fig. 3:**
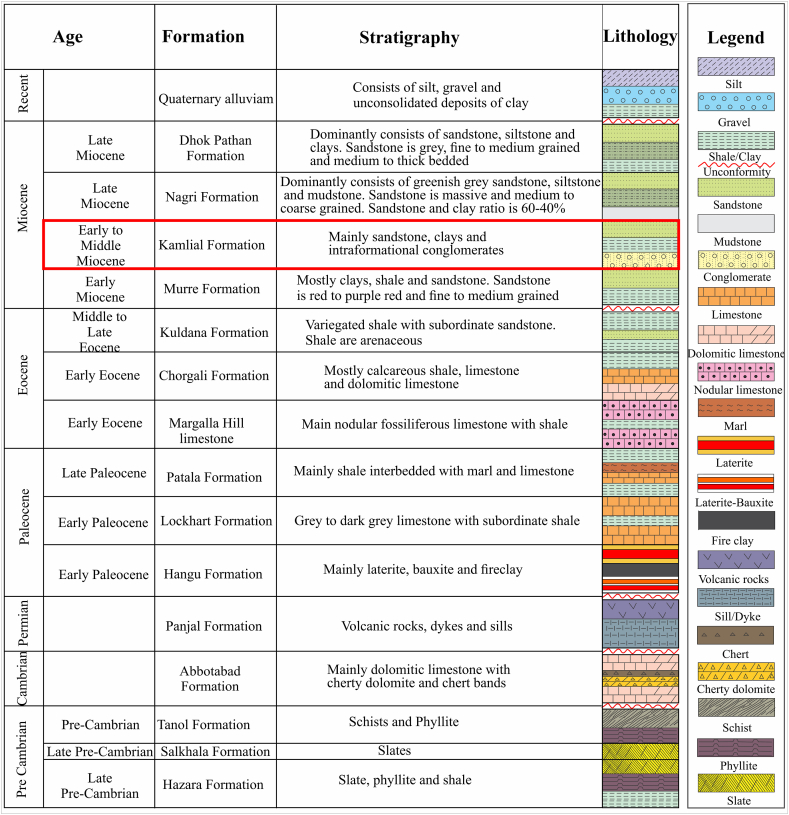
Litho-stratigraphy of the Muzaffarabad and Dhirkot areas. Red rectangle shows the study area. The stratigraphy has been modified after [17,34,35]. (For interpretation of the references to color in this figure legend, the reader is referred to the Web version of this article.)

A Pre-Cambrian to recent stratigraphic sequence is exposed in Muzaffarabad and Dhirkot (Table 1). The stratigraphic details of the area are present in (Fig. 3) and are discussed in detail in the following sub-sections.

### 3.1 Hazara Formation

Hazara Formation has been named by Refs. [8,38] based on the "Slate series of Hazara" and "Hazara slate Formation". [39,40] named the Formation "Hazara Group" after [8] incorporated the Tanawal Formation into it. The Hazara Formation is the oldest rock Formation in Muzaffarabad and surrounding areas. Phyllite, slate, and shale make up the Hazara Formation. Furthermore, sandstone, carbonates, and graphite layers are present [8]. A slate or phyllite's fresh surfaces are appear green to dark green and black. In contrast, a slate or phyllite with a weathered surface are appear rusty brown or dark green. There are several areas in Muzaffarabad well exposed to it, including Chatter, Gojra, and Lohar Gali. A fault exists in Muzaffarabad at the intersection of the Hazara and Murree Formations.

### 3.2 Salkhala Formation

Salkhala Formation was named by Ref. [8]. Locality of type is Salkhala, near the Kishanganga River in Kashmir. This Formation is composed of various types of rock, including marble, quartzite, garnet-biotite, quartz schist, and talc schist. The Salkhala Formation can be seen at the apex of the Syntaxis in the Hazara region. There is a thinned-out Formation section near Balakot. Marble, graphite schist, quartz schist, and quartz-feldspathic gneiss are major components of the Hazara Formation. To the south of Kashmir, schistose rocks form an extremely folded Formation. Salkhala beds were identified by Ref. [41] as part of the Sharda group north of Salkhala Village, across the Neelum River.

### 3.3 Tanol Formation

[42] believes rocks of the Formation are members of the "Tanol Group". The rock is known as 'Tanol quartzite', according to Ref. [38]. [39,40,43] named the Tanol Formation. It is composed mainly of quartzose schists, quartzites, and schistose conglomerates. It is exposed along the western edge of the Hazara Kashmir Syntaxis and along the southern and southeastern borders of the Mansehra Granite Formation. Kashmir has well exposed examples of the Formation in the Tithwal and Loath areas of the Neelum Valley as well as the Cham areas of the Jhelum Valley. These rocks are intruded by Jura granite gneiss and Cham granite gneiss. [44] dated the Formation to the Late Precambrian.

### 3.4 Abbottabad Formation

[8] Refer to a dolomite, quartzite, and phyllite Formation in southern Hazara as the Abbottabad Formation. In the Muzaffarabad region, a thick layer of quartzose sandstone overlies the basal conglomerates, followed by a layer of dolomite. At the Yadgar section in Muzaffarabad, stromatolites are found in the Abbottabad Formation. Abbottabad Formation is characterized by chopped boards. Based on fossil evidence, the Formation is dated to the Early Cambrian [45].

### 3.5 Panjal Formation

[46] named the Panjal Volcanic Series. [8] says the entire rock unit consists of two parts, the Panjal Formation, and the Agglomerate slate. The Panjal volcanic is described by Ref. [14] as occurring between the Panjal Thrust and the Main Boundary Thrust. Green schist facies are formed by basaltic lava flows. Panjal volcanic rocks are found in Muzaffarabad, Kahuta, Srinagar, and Kaghan. [8] says volcanic greenstone exhibits weakly developed schistosity parallel to primitive layering. In the Lamnian and Batangi areas of the Jhelum Valley, as well as the Nausseri area of the Neelum Valley, Muzaffarabad, these rocks are exposed along the MBT. The Panjal Formation is dated from the Carboniferous to the Permian in western Kashmir, and from the Triassic to the Carboniferous in eastern Kashmir [8].

### 3.6 Hangu Formation

[47] described the Hangu shale and Hangu sandstone from the Kohat area [45] as the Hangu Formation. In the Kohat-Potwar region, it is called "Mari Limestone" by Refs. [39,40]. The type locality is Fort Lockhart. Medium-bedded, fine-grained, thick sandstone is present in the area. Coal, bauxite, and dark grey shale comprise the Hangu Formation. The Hangu Formation also contains fossils such as Operculina and Lochartia Haimei [35]. The Formation is visible along the Neelum Valley road in Muzaffarabad. There is an unconformable contact between Hangu Formation and the Abbottabad Formation at the lower level. In contrast, the conformable contact between the Hangu Formation and the Patala Formation is at the upper level. [[48], [49], [50]] says the Hangu Formation dates from the Early Paleocene.

### 3.7 Lockhart Formation

"Lockhart Limestone" refers to Paleocene limestone found in Kohat. This name has been expanded by the Stratigraphic Committee of Pakistan to refer to the same units in other parts of Kohat-Potwar and Hazara. It is possible to observe the Formation well along the Neelum Valley road in the Muzaffarabad area. The Lockhart Limestone consists of three types of limestone: dark grey to black, fine-to medium-grained, nodular limestone, and fossiliferous limestone with medium to high tenacity. This formation dates to the Paleocene [47]. The Lockhart limestone and the Hangu Formation are in conformance with each other. Patala Formation and upper contact are separated by a transitional zone.

### 3.8 Patala Formation

The term Patala Shale was originated by Ref. [48]. It was renamed the Patala Formation by Pakistan's Stratigraphic Committee [45]. It is primarily composed of shale and marl, with subordinates' limestone and sandstone. Shale varies in colour from greenish brown to buff. The Neelum Valley Road exposes a section of the Neelum Valley Formation. The Formation is characterized by widespread fossilization. The Hazara region contains fossils such as Globorotalia, Triloculina, and Assilina, according to Refs. [39,40]. Lockhart's lower contact is transitional. There is an unconformity in the upper contact with Margalla Hill Limestone. It is believed that the Formation dates back to the Late Paleocene [17].

### 3.9 Margalla Hill Limestone

Margalla Hill Limestone has been officially named by the Stratigraphic Committee of Pakistan [45]. It is exposed in the Kohat-Potwar Basin and the Hazara region. These rocks are exposed on the Neelum Valley road in Muzaffarabad. [[38], [39], [40],^42^,^51^], have studied these rocks. In this area, limestone is the dominant rock type, while shale and marl are the subordinate rocks. On a fresh surface, limestone appears grey, while on a weathered surface, it appears yellowish grey. The limestone is fine-to medium-grained with nodular grains and medium to thick bedded limestone. The Formation is believed to have formed during the Early Eocene. It is unconformable to the Patala Formation at the lower contact, whereas the Chorgali Formation is conformable at the upper contact.

### 3.10 Chorgali Formation

The Chorgali Formation has been named by Ref. [52] and is accepted by the Pakistani Stratigraphic Committee. A well-known type locality for the Formation is Chorgali Pass within the Khair-e-Murat range [39,40]. The Formation is composed of shale and limestone. It consists of limestone, dolomitic limestone, and shale in the Kashmir region. This Formation can be found along the Neelum Valley Road.

### 3.11 Kuldana Formation

The Kuldana Formation is recognized by the Pakistani Stratigraphy Committee. This Formation consists of type sections located near Kuldana, north of Murree Hill Station. This is in the Hazara district near Murree Hill Station. Rocks from the Kuldana Formation can be seen along the Neelum Valley road. Sandstone, limestone, conglomerates, and marls are found in this region. The Formation dates from the Early to Middle Eocene, according to Ref. [17].

### 3.12 Murree Formation

The Stratigraphy Committee of Pakistan has designated the Murree Formation. It is named after the Murree Hills in Rawalpindi district. The Formation is widely exposed in the Kohat-Potwar region. In general, it is also found in the Kashmir region. In various parts of Kashmir, including the Muzaffarabad and Bagh districts, medium-to coarse-grained sandstone, siltstone, and shale are found. Sandstone has a variety of colors, including purple, reddish, and grey colors. Sandstone is hard and compact. Fossilization is poor in the Formation. The Formation was formed during the Early Miocene [17].

### 3.13 Kamlial Formation

Kamlial beds are now known as the Kamlial Formation, according to the Stratigraphic Committee of Pakistan. In addition to greenish grey and blueish sandstone that is medium-to coarse-grained, it also contains crystalline purple greenish and yellowish shale. It also contains yellow and purple intra formational conglomerates. Due to its high mineral content, spheroidal weathering, and dominance of tourmaline over epidote, it differs from the Murree Formation.

### 3.14 Nagri Formation

The Pakistani Stratigraphic Committee has recognized the Nagri Formation. Clay and conglomerates are also present in the Formation along with sandstone. Colored greenish grey, the sandstone is medium to coarse in grain size, cross-bedded, and massive. There are places where the sandstone is bluish grey, dull red with "salt and pepper" patterns, calcareous, and moderately or poorly cemented. Clay is generally sandy or silty, chocolate brown or reddish grey, and pale orange in color, with proportions varying from section to section. The thickness and composition of conglomerate beds vary considerably. Kohat-Potwar region has igneous rocks and Eocene limestone pebbles. Mang is the area in where the site is located. [17]) states that the Nagri Formation's age ranges from the Late Middle to the Late Miocene, while that of the Kohat-Potwar Formation is Early Pliocene in age.

### 3.15 Dhok Pathan Formation

Dhok Pathan was named by Ref. [53] after the village of Dhok Pathan in the district of Attock. According to Ref. [54], the Pakistani Stratigraphic Committee accepts the name Dhok Pathan Formation. Several sandstone beds have been exposed in the Jassa Pir Mang area, primarily from the Dhok Pathan Formation. In the Dhok Pathan Formation, sandstone and clay are found in approximately equal proportions, indicating that they were deposited cyclically. A medium to thick sandstone bed is interbedded with conglomerate beds between the sandstone beds. Quartz grains vary from being rounded to angular and coarse in texture. Other minerals include muscovite, biotite, feldspar, epidote, tourmaline, and pink garnet. There are gradational contacts between the Dhok Pathan Formation and the underlying Nagri Formation. The Dhok Pathan Formation dates from the Late Miocene [45].

### 3.16 Quaternary alluvium

In quaternary alluvium, boulders are rounded to subrounded, along with cobbles, pebbles, and gravels of different rocks, including metamorphic and igneous materials.

## 4 Materials and methods

### 4.1 Field data collection

**Fig. 4:**
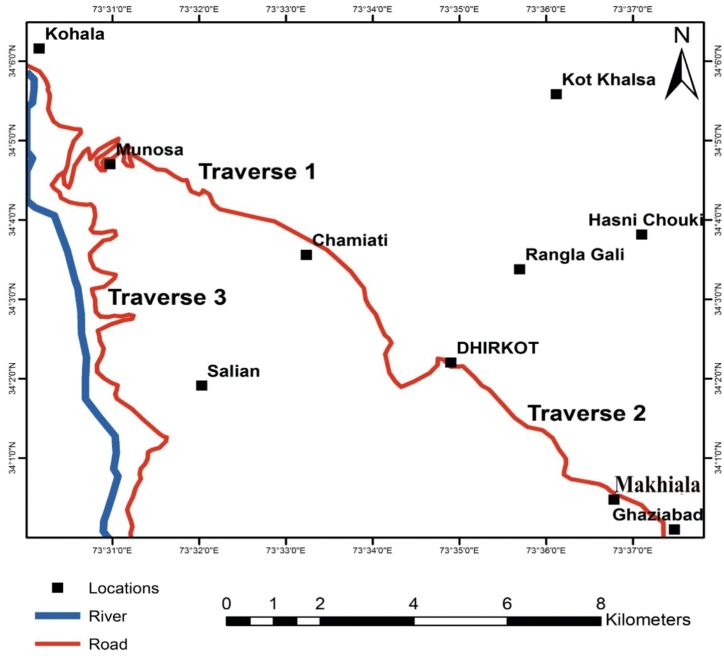
Traverse map of the study area.

The fieldwork was conducted in Bagh district of Azad Jammu and Kasmir area. We collected 36 samples from Kamlial Formation of the Miocene age in three sections with the latitude between 34°1′0"N to 34°6′0"N and longitude between 73°31′0"E to 73°37′0"E (Fig. 2, Fig. 4). During the filed, rock samples were located using topographic sheets and a Brunton compass was used to measure the dip and strike of the beds. All fresh samples were collected for petrographic analysis from the outcrops with a geological hammer. All the relevant field features were also noted. Fine grain size, texture of the rocks units was observed through a hand lens (10x) while megascopic sedimentary structures were observed through the naked eye. To capture photographs of prominent features in the rock, a high-resolution camera was utilized. During the fieldwork, traverses were made along and across the regional strikes. The thin sections of these rock samples were prepared in the laboratory of Geosciences, Islamabad.

### 4.2 Laboratory analysis

A total of 36 thin sections were prepared at the Laboratory of Geosciences, Islamabad. A polarizing microscope was employed in the petrography laboratory of the University of Azad Jammu and Kashmir to examine thin sections. It was also used to quantify mineral grains using point counting techniques. Quantitative estimates of the mineral composition were obtained with a point count of 300 grains on each thin section. Petrographical properties of the rock samples such as grain fabric, contact, textural attributes, mineralogical composition, and the cement types were examined. Based on a classification scheme, the provenance, diagenesis and depositional environment of the sandstones were evaluated. The model mineralogy of the total sample data was plotted on a QAF triangle [55] and the rocks were classified based on the model mineralogy. Moreover, geological and traversing maps of the study area were prepared using various software applications such as Arc GIS 10.5, Corel Draw in the computer laboratory of the University of Azad Jammu and Kashmir.

## 5 Results

### 5.1 Petrographic description

**Fig. 5:**
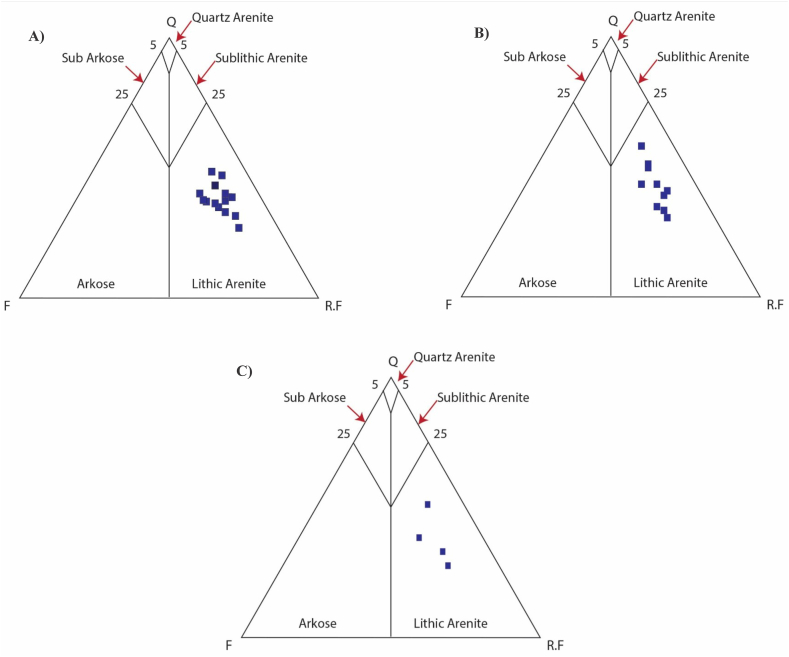
Modal mineralogy data of sandstone of Kamlial Formation. (A): From Kohala to Dhirkot section. (B): Dhirkot to Ghaziabad section, and (C): Khapaddar to Kohala sections plotted on QFL diagram for classification of field boundaries [55].

**Fig. 6:**
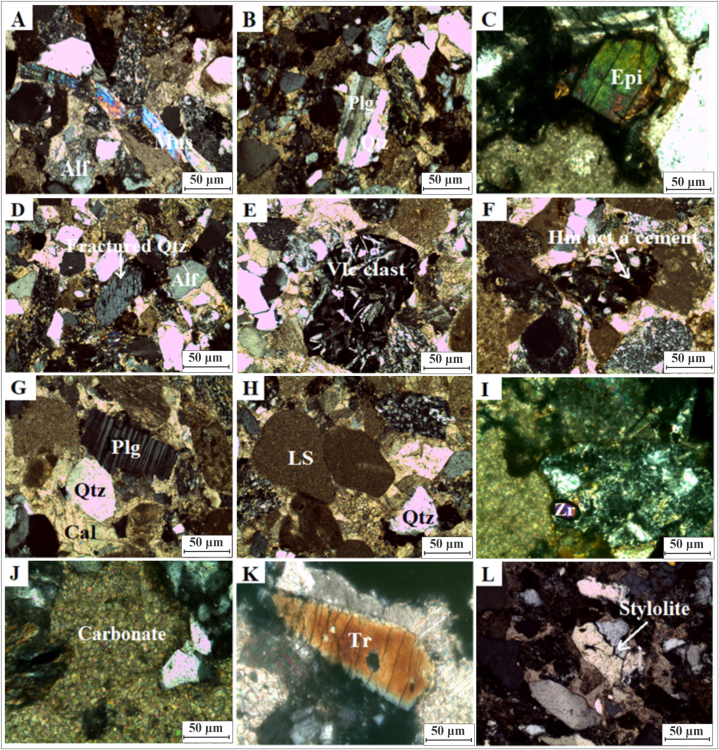
(A): Photomicrograph of lithic arenite shows deformation in muscovite (Crossed Nicols 10x). (B): Photomicrograph of lithic arenite shows planner and suture contacts in quartz grain (Crossed Nicols 10x). (C): Photomicrograph is showing grain of epidote surrounded by hematite/ferruginous cementing material (Crossed Nicols). (D): Photomicrograph of lithic arenite shows the grain of quartz which is highly fractured (Crossed Nicols 10x). (E): Photomicrograph shows volcanic fragment which is surrounded by calcite (Crossed Nicols 10x). (F): Photo micrograph of lithic arenite shows hematite which is acting cementing material in the sandstone clast (Crossed Nicols 10x). (G): Photo micrograph shows quartz, plagioclase and cementing material which is carbonate (Crossed Nicols 10x). (H): Photo micrograph shows the grains of limestone in the lithic arenite (Crossed Nicols 10x). (I): Photomicrograph of lithic arenite is showing zircon (Crossed Nicols 40x). (J): Photomicrograph of lithic arenite shows carbonate acts as cementing material (Crossed Nicols 40x). (K): Photo micro graph shows the elongated tourmaline (Crossed Nicols 40x). (L): Photo micrograph shows stylolite feature between grains of quartz (Crossed Nicols 10x). Alf = alkali feldspar, Plg = plageoclase, Qtz = quartz, Epi = epidote, Vlc clast = volacano clast, Hm = hematite, Cal = calcite, LS = lime stone, Zr = zircon, Tr = tourmaline.

**Fig. 7:**
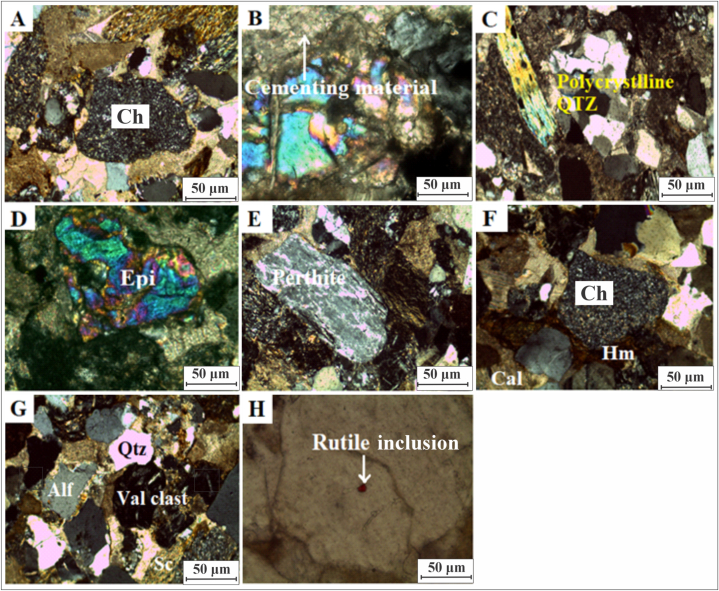
(A): Photomicrograph shows clast of chert. (Crossed Nicols 10x). (B): Photomicrograph of lithic arenite shows the cementing material (Crossed Nicols 40x). (C): Photomicrograph shows polycrystalline quartz (Crossed Nicols 10x). (D): Photomicrograph is showing epidote (Crossed Nicols 40x). (E): Photomicrograph shows perthite (Crossed Nicols 10x). (F): Photomicrograph shows clast of chert surrounded by hematite and calcite cements (Crossed Nicols 10x). (G): Photomicrograph of lithic arenite shows quartz, volcanic clast, and schist (Cross Nicol 10x). (H): Photo micro graph shows inclusion of rutile in grain of quartz (PPL 40x). Ch = chert, Qtz = quartz, Epi = epidote, Hm = hematite, Cal = calcite, Alf = alkali feldspar, Val clast = volcano clast, Sc = schist.

**Fig. 8:**
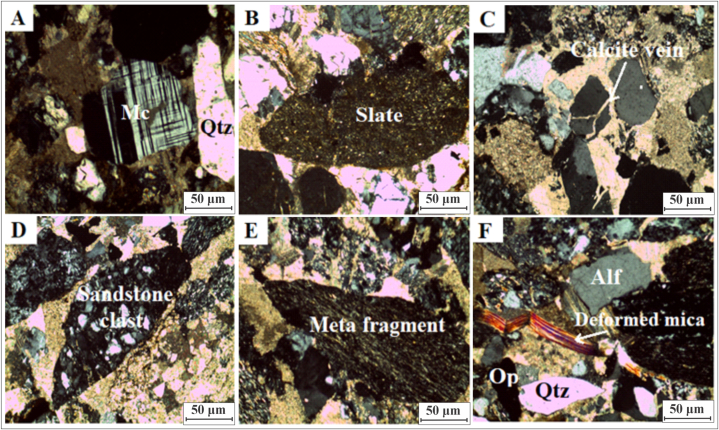
(A): Photomicrograph of lithic arenite shows microcline in lithic arenite (Crossed Nicols 10x). (B): Photo micro graph shows slates (Crossed Nicols 10x) (C): Photomicrograph showing calcite vein in quartz (Crossed Nicols 10x). (D): Photomicrograph of lithic arenite shows clast of sandstone (Crossed Nicols 10x). (E): Photomicrograph of lithic arenite shows a grain of biotite schist in lith arenite (Crossed Nicols 10x). (F): Photomicrograph shows of lithic arenite shows deformed muscovite (Crossed Nicols 10x). Mc = microcline, Qtz = quartz, Alf = alkali feldspar, Op = opaque.

**Fig. 9:**
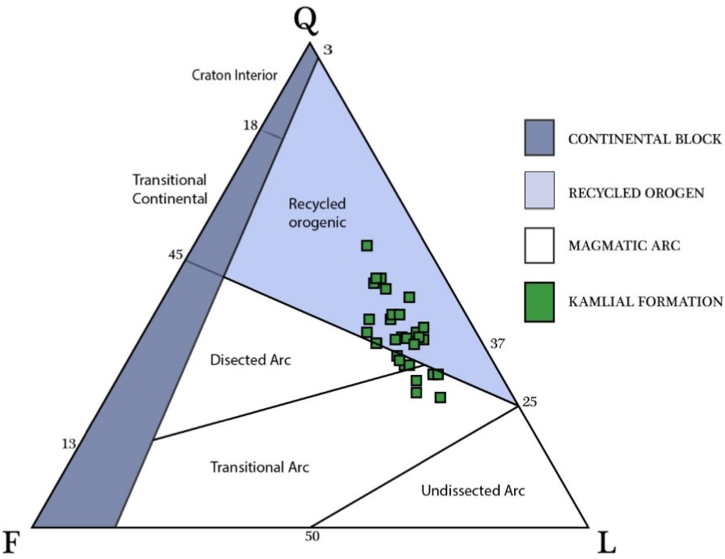
The quartz, feldspar and lithics (rock fragments) data of the sandstone is plotted on the provenance discrimination diagram of [56].

We selected 36 samples from three sections which including (1) Kohala to Dhirkot section (2) Dhirkot to Ghaziabad (3) Khapaddar to Kohala section (Fig. 2, Fig. 4). We analyzed the thin sections from these samples for different petrographic characteristics. Microscopically and based on petrographic QFL data plotted on diagrams of sandstone classifications, the Kamlial Formation sandstone falls into the lithic arenite group (Fig. 5). Sandstone grains are predominantly angular to sub-angular and rounded to sub-rounded. There are very few stretched quartz grains. Most quartz grains are monocrystalline and show uniform extinction, while some are polycrystalline. The sandstone contains alkali feldspars as microcline and perthite as well as plagioclase with albite twining. Tourmaline, epidote, zircon, biotite, muscovite, and rutile are some of the accessory minerals found in rock samples (Fig. 6, Fig. 7, Fig. 8). Most of the cementing material found during the study was carbonate. However, they also contain calcite veins. Sandstone of the Kamlial Formation contains rock fragments such as interformational and intraformational clasts. Interformational clasts in sandstone of Kamlial Formation include metamorphic, igneous, and sedimentary rocks, including slates, biotite schists, quartzites, volcanic clasts, chlorite schists, limestones, and intraformational clasts in sandstone of Kamlial Formation consists of both siltstone and sandstone clasts. Tourmaline, epidote, zircon, biotite, muscovite, and rutile are some of the accessory minerals found in the rocks. Most of the cementing material is carbonate. To determine the provenance of the sandstone of the Kamlial Formation, quartz, feldspar, and rock fragments (QFL) data were analyzed. QFL data were plotted on the sandstone provenance discrimination diagram developed by Ref. [56]. QFL data from the Kamlial Formation fall within the field of recycled orogens (Fig. 9).

**Fig. 10:**
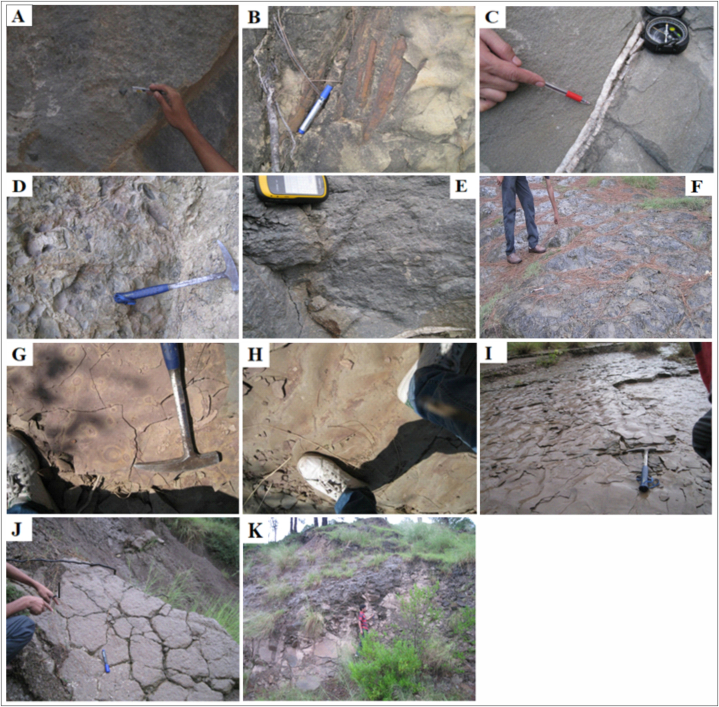
(A): Field photograph showing quartzite clast in sandstone of Kamlial Formation near Ghaziabad. (B): Photograph shows petrified wood is found in Kamlial Formation. (C): Photograph showing calcite vein in sandstone of Kamlial Formation. (D): Photograph showing load casts in Kamlial Formation. (E): Photograph showing intraformational microconglomerates in sandstone of Kamlial Formation. (F): Photograph showing spheroidal weathering in Kamlial Formation. (G): Photograph showing calcite concretion in Kamlial Formation. (H): Photograph showing iron leaching in Kamlial Formation. (I): Photograph showing ripple marks in Kamlial Formation. (J): Photograph showing mud cracks in the Kamlial Formation. (K): Photograph showing breccia in the field area.

In the outcrop, the Kamlial Formation consists of bluish and grey sandstone that is medium-to coarse-grained and compacted. In addition, geological features (Fig. 10) such as, load casting, intraformational conglomerates, and iron leaching (Fig. 10D and E,H), quartzite clasts, and petrified wood were observed during field work (Fig. 10A and B). Generally spheroidal weathering (Fig. 10F) and a heavy mineral content including tourmaline and epidote distinguish it from the Murree Formation. In the Kamlial Formation (Fig. 10G), calcite concretions are also found. Calcite fills fractures at various locations (Fig. 10C).

#### 5.1.1 Petrography of sandstone from Kohala to Dhirkot section

The rocks exposed in the Kohala to Dhirkot section are Kamlial sandstone. The sample location map shows the location of the samples collected in this study (Fig. 4). During field work in this section, 16 sandstone samples were collected for petrographic analysis. According to the modal mineralogical composition of the thin sections, all samples are lithic arenites (Fig. 5A). The petrographical study of the studied samples indicates that quartz is the main constituent of sandstone. In total, the quartz content ranges from 17 to 31% (Table 2). There are different shapes of quartz, including angular, subangular, rounded, and subrounded. Monocrystalline quartz grains exhibit uniform and non-uniform extinction patterns. It is uncommon that quartz grains are polycrystalline. Quartz contains stylolite features (Fig. 6L). The stylolite feature was present between quartz grains. Various contacts between the grains were determine including point, planner, and concave-convex contacts. (Fig. 6B) illustrates lithic arenite long and suture contacts in quartz grain (Crossed Nicols 10x). Some quartz grains have been fractured due to intense tectonic activity, as evident in the quartz grain highly fractured (Fig. 6D).

**Table 2:** Modal mineralogical data of samples in Kohala to Dhirkot section.

Samples																		
Samples ID			2	4	5	6	7A	7B	9	10	11	12	13	14	15 A	15B	16	17
Model mineralogy																		
Quartz	Monocrystalline		19	17	23	27	23	19	20	18	29	20	27	24	17	22	14	19
	Polycrystalline		2	3	5	2	4	2	3	4	2	5	4	2	3	1	3	2
Feldspar	Orthoclase		1	1	1	2	–	–	3	–	–	1	1	1	1	–	–	2
	Microcline perthite		4	6	3	2	3	4	4	3	3	2	2	3	2	4	2	2
	Plagioclase		5	3	4	4	4	6	5	5	3	4	4	4	5	7	5	4
Rock fragments	Igneous	Plutonic		–	–	–	–	–	–	–	–	–	–	–	–	–	–	–
		Volcanic	7	6	6	7	5	7	6	11	7	10	6	3	5	5	7	5
	Metamorphic	Quartzite	1	1	2	3	3	2	2	1	2	1	1	2	2	2	2	2
		Gneisses	1	–	–	1	–	1	2	–	1	1	–	–	1	–	1	2
		Slate	1	1	2	2	3	3	–	1	1	2	1	1	2	1	2	–
		Phyllite	–	–	–	–	1	–	–	2	–	–	1	1	–	1	–	–
		Mica schist	6	3	6	8	7	5	5	6	5	7	5	4	10	4	11	8
		Chlorite schist	1	1	2	4	2	3	3	2	1	2	2	2	3	2	5	5
	Sedimentary	Sandstone	5	4	5	6	7	6	5	4	5	4	6	5	7	6	6	5
		Siltstone	4	3	3	4	6	2	3	4	4	4	2	6	4	3	2	4
		Limestone	2	1	2	2	2	1	2	2	3	–	1	2	2	2	1	2
		Dolomite	–	–	–	–	–	–	–	–	–	–	–	–	–	–	–	–
Accessory minerals	Muscovite		3	4	3	2	2	3	5	4	3	2	3	3	2	4	3	4
	Biotite		2	3	2	2	2	2	4	3	2	3	2	2	2	2	2	2
	Tourmaline		1	2	2	2	1	1	1	2	1	1	1	2	1	1	1	1
	Zircon		–	–	Tr	–	–	–	Tr	–	Tr	–	–	1	–	–	–	–
	Hornblende		1	1	1	1	1	1	1	–	1	–	–	1	1	2	1	1
	Epidote		1	3	1	1	1	2	1	1	1	1	–	1	1	1	2	1
	Sericite		Tr	1	–	Tr	Tr	Tr	Tr	–	Tr	–	1	–	–	2	2	1
	Apatite		Tr	–	Tr	–	–	Tr	–	–	–	–	–	–	–	–	1	–
	Chlorite		1	2	2	1	2	1	2	2	1	2	3	2	2	3	1	2
	Hematite		3	4	2	–	2	1	–	2	4	3	2	2	2	3	1	–
Cements	Calcite		17	16	13	11	12	16	16	15	11	16	16	17	16	15	14	18
	Quartz		–	1	–	–	1	2	–	1	1	1	–	–	–	–	2	–
	Hematite		4	3	4	1	2	2	4	1	3	2	3	4	2	2	2	3
	Silica		–	1	–	–	–	1	–	–	–	–	1	–	–	–	1	–
	Clay		–	1	–	–	–	1	–	1	–	–	1	–	–	–	1	–
Opaque minerals			5	7	3	2	2	3	3	2	3	2	3	3	5	4	3	3
Matrix			2	1	2	2	1	2	3	3	2	4	1	2	2	1	2	2
Total			100	100	100	100	100	100	100	100	100	100	100	100	100	100	100	100

Feldspar content ranges from 6 to 11% (Table 2). Most of feldspar is altered into clay minerals and sericite. There are plagioclase grains (Fig. 6G), microcline perthite grains, and orthoclase grains. The lithic arenite deformation was noted in muscovite (Fig. 6A). In addition to quartz and feldspar the Kamlial Formation at this station also contains rock fragments of igneous, sedimentary, and metamorphic origin. Rock fragments in thin sections range from 20 to 37% (Table 2). Sandstone is composed of fragments of the Panjal volcanics (Fig. 6E). The (Fig. 6E) represents the volcanic fragment surrounded by calcite. A variety of metamorphic rock fragments were present, including slates, chlorite schists, biotite schists, and quartzites. Sandstone, siltstone, and limestone are the most prevalent sedimentary clasts (Fig. 6H,J). Carbonates of lithic arenite serve as cementing materials (Fig. 6J). The cementing material is primarily calcite accounts for 8–23% of the cementing material (Table 2). Hematite and silica also serve as cementing materials (Fig. 6F). Biotite, muscovite, zircon inclusions in quartz, epidote, tourmaline, and opaque minerals are observed as accessory minerals (Fig. 6 C,I and K). In Fig. 6C, the epidote grain is surrounded by ferruginous/hematite cementing material, while lithic arenite (Fig. 6I) is exhibiting zircon, and Fig. 6K is the elongated tourmaline. Most of clasts are angular or sub-angular, while some are rounded or sub-rounded.

#### 5.1.2 Petrography of sandstone from Dhirkot to Ghaziabad section

In this section, Kamlial Formation rocks are exposed on the road section between Dhirkot and Ghaziabad. The petrographic study of this section was conducted on ten sandstone (lithic arenites) samples (Fig. 5B). Quartz constitutes a large proportion of the minerals in this section's as determined by petrographic analysis of samples. Sub-angular to angular quartz grains exhibit planar and suture contacts and are highly fractured and stretched. Additionally, there are more monocrystalline quartz grains than polycrystalline (Fig. 7C) quartz grains. Undoluse extinction is present in some quartz grains, however, it is not present in other minerals.

The quartz content ranged from 19 to 42%, while feldspar content ranged from 6 to 10%. Table 3 outlines the details of mineralogical data for samples from Dhirkot to Ghaziabad. Plagioclase exhibited albite twinning, and orthoclase exhibited carlsbad twinning. The feldspar has been highly altered to sericite. Plagioclase, microcline perthite (Fig. 7E), and orthoclase make up most of the grains. The (Fig. 7D,E) represent epidote and perthite respectively. In the sandstone, lithic fragments of the Panjal volcanics are found. Metamorphic rock fragments include slate, phyllite, chlorite schist, biotite schist, and quartzite (Fig. 7A,G). There was the rock fragment of chert in (Fig. 7 A,F) and surrounded by ferruginous and calcite cements (Fig. 7F). The lithic arenite with quartz, volcanic clast and schist was represented in (Fig. 7G). Sandstone, siltstone, and limestone constitute most sedimentary clasts (Fig. 7F). Cementing materials are mainly composed of calcite (Fig. 7B), accounting for 14–23% (Table 3). Hematite and silica, on the other hand, serve as cementing materials as well. The Kamlial sandstone petrography also exhibits rutile inclusion (Fig. 7H).

**Table 3:** Modal mineralogical data of samples in Dhirkot to Ghaziabad section.

Samples												
Samples ID			18	19	21	24	25	26	28	29	30	31
Model mineralogy												
Quartz	Monocrystalline		22	17	17	20	19	38	25	23	27	25
	Polycrystalline		4	2	2	4	3	4	2	2	2	3
Feldspar	Orthoclase		–	2	–	2	1	2	–	1	1	–
	Microcline perthite		2	5	2	3	4	2	3	3	2	2
	Plagioclase		4	4	7	5	6	4	5	4	4	5
Rock fragments	Igneous	Plutonic	–	–	–	–	–	–	–	–	–	–
		Volcanic	5	8	7	2	7	4	4	6	9	4
	Metamorphic	Quartzite	2	3	1	1	2	1	2	2	1	2
		Gneisses	1	1	1	2	–	1	–	–	1	–
		Slate	1	2	2	1	2	2	1	1	–	1
		Phyllite	1	–	–	1	–	–	1	1	–	–
		Mica schist	6	8	7	5	7	3	6	4	7	6
		Chlorite schist	1	1	2	2	2	1	1	2	1	–
	Sedimentary	Sandstone	6	6	5	4	5	4	5	6	5	3
		Siltstone	5	5	2	2	3	5	4	5	4	3
		Limestone	3	3	2	2	2	1	2	4	1	2
		Dolomite	–	1	–	–	–	–	–	–	–	–
Accessory minerals	Muscovite		2	2	3	5	3	2	3	2	2	2
	Biotite		2	1	3	4	2	2	4	1	2	2
	Tourmaline		1	1	1	2	2	1	1	1	Tr	1
	Zircon		–	–	–	–	Tr	–	–	Tr	–	–
	Hornblende		–	1	–	1	1	1	–	–	Tr	–
	Epidote		Tr	1	1	Tr	1	1	1	–	–	1
	Sericite		Tr	–	–	Tr	–	–	–	Tr	Tr	–
	Apatite		–	–	–	Tr	1	–	–	–	–	–
	Chlorite		1	1	1	2	2	1	2	Tr	–	1
	Hematite		3	1	5	1	2	–	2	2	–	2
Cements	Calcite		19	16	19	18	14	14	15	22	23	20
	Silica		–	Tr	Tr	–	–	1	1	Tr	–	–
	Hematite		2	2	4	3	3	1	3	4	1	1
	Clay		–	–	–	1	–	–	–	–	–	–
Opaque minerals			4	2	3	5	3	3	3	2	2	2
Matrix			2	1	2	2	2	1	3	1	2	2
Total			100	100	100	100	100	100	100	100	100	100

#### 5.1.3 Petrography of sandstone from Khapaddar to Kohala section

The rocks exposed along the Bandi to Kohala road section are part of the Kamlial Formation. For petrographic studies, four samples of Kamlial sandstone (lithic arenites) were collected during field work (Fig. 5C). Table 4 represents the modal mineralogical data of samples obtained from the Khapaddar to Kohala road section. Petrographic analysis of these samples revealed that quartz accounts for 18–34% in composition. Moreover, some quartz grains exhibit undulatory extinction, while others are subangular to angular. In addition to being slightly stretched, quartz grains exhibit point, long, concavo-convex, and suture contacts. Monocrystalline grains are more abundant than polycrystalline grains. A stylolite feature is seen between quartz grains.

**Table 4:** Modal mineralogical data of samples in Khapaddar to Kohala road section.

Samples						
Samples ID			32	33	34	36
Model mineralogy						
Quartz	Monocrystalline		22	20	30	15
	Polycrystalline		4	3	4	3
Feldspar	Orthoclase		–	–	–	2
	Microcline perthite		6	4	3	2
	Plagioclase		5	7	5	4
Rock fragments	Igneous	Plutonic	–	–	–	–
		Volcanic	7	5	4	5
	Metamorphic	Quartzite	2	2	2	2
		Gneisses	–	–	1	2
		Slate	2	1	2	–
		Phyllite	–	1	1	–
		Biotite schist	5	4	3	8
		Chlorite schist	2	2	1	5
	Sedimentary	Sandstone	5	6	6	5
		Siltstone	4	3	2	4
		Limestone	3	2	1	2
		Dolomite	1	–	–	–
Accessory minerals	Muscovite		2	4	2	4
	Biotite		2	2	2	2
	Tourmaline		1	2	1	1
	Zircon		Tr	–	–	–
	Hornblende		1	2	Tr	1
	Epidote		1	–	0	1
	Sericite		Tr	Tr	Tr	Tr
	Apatite		–	–	Tr	–
	Chlorite		2	1	1	2
	Hematite		3	5	1	–
Cements	Calcite		13	17	14	19
	Silica		2	–	3	–
	Hematite		2	2	2	3
	Clay		–	Tr	–	Tr
Opaque minerals			2	4	3	3
Matrix			1	1	2	2
Total			100	100	100	100

(Fig. 8A) shows a microcline and (Fig. 8F) shows deformed muscovite. The feldspar percentage ranges from 8 to 11% (Table 4). It contains plagioclase, orthoclase, and microcline. In the sandstone, rock fragments are composed of Panjal volcanics, sedimentary, and metamorphic rocks. These fragments include slates (Crossed Nicols 10x) (Fig. 8B), sandstone (Fig. 8D), and biotite schist grains (Crossed Nicols 10x) (Fig. 8E). The cement contains approximately 13–19% calcite, as shown in Table 4. Hematite and silica are also used as cementing materials. The (Fig. 8C) represents calcite vein in quartz (Crossed Nicols 10x).

### 5.2 Field features of the study area

The study area is comprised of the Kamlial Formation. The Kamlial Formation sandstone is exposed along the Kohala to Dhirkot section, the Dhirkot to Ghaziabad section, and the Khapaddar to Kohala road sections. The Kamlial Formation contacts the Muree Formation at its lower end. Kamlial sandstone has a bluish-grey color. In the study area near Ghaziabad, quartzite clasts were observed during field work at latitude N34°01′43ℙ and longitude E73°35′56ℙ (Fig. 10A). In addition, petrified wood was found in the Kamlial Formation on the Dhirkot to Ghaziabad Road section at latitude N34°01′43ℙ and longitude E73°35′56ℙ. The (Fig. 10B) shows petrified wood found in Kamlial Formation. Calcite veins are common in the sandstone of Kamlial Formations at latitude N34°01′43ℙ and longitude E73°35′56ℙ. The (Fig. 10C) represents calcite vein in the sandstone of Kamlial Formation. Load casts are formed at the base of denser layers such as sand, which are layered over denser layers such as silt and shale. The load casts demonstrate different depositional environments, including fluvial and shallow-marine environments. Load casts are present at different locations in the research area at latitude N34°00′80ℙ and longitude E73°36′14ℙ. The (Fig. 10D) displays load casts present in the Kamlial Formation.

The Kamlial Formation in the Dhirkot to Ghaziabad section contains conglomerates at latitude N34°01′43ℙ and longitude E73°35′56ℙ. Conglomerate found within the Formation is called an intra formational micro conglomerate (Fig. 10E). Moreover, the study area along the Kohala to Dhirkot road exhibits spheroidal weathering (Fig. 10F). Among the other Formations in the research area, the Kamlial Formation is characterized primarily by spheroidal weathering. Furthermore, they demonstrate calcite concretion (Fig. 10G) and iron leaching (Fig. 10H). Sedimentary structures observed in the field area include the following: Ripple marks in (Fig. 10I) located at latitude N34°06′94ℙ and longitude E73°30′61ℙ and mud cracks are represented in (Fig. 10J). A fault breccia located at latitude N34°06′00ℙ and longitude E73°30′56ℙwas found in the study area (Fig. 10K).

## 6 Discussion

### 6.1 Provenance of the sandstone

Based on petrographic analysis, Kamlial Sandstone was classified as lithic arenite (Fig. 5). Petrographically, Kamlial sandstone contains quartz, rock fragments, and feldspar. In the lithic arenite of the Kamlial Formation, siltstone and sandstone are intraformational rock fragments (Table 2, Table 3, Table 4). Some thin sections have polycrystalline quartz with undulatory extinction, which indicates that the source area was metamorphic [[57], [58], [59], [60]]. However, some samples contain monocrystalline quartz that does not have undulose extinction, which indicates granitic, preexisting sedimentary rocks and volcanic rocks were present in the source area [[57], [61], [62], [63]].

In the study area, quartz may be derived from metamorphic, igneous, and pre-existing sandstone. Quartz grains with angular to subangular faces demonstrate that the source area was close to the depositional site. In contrast, quartz grains with rounded faces indicated that the source area was far from the depositional site. Moreover, roundness was also indicative of long-distance transportation and several cycles of erosion and deposition. Metamorphic source rock can also be identified as metamorphic fragments in the rocks [64]. Feldspar in the Kamlial Formation is dominated by minor orthoclase, plagioclase, perthite and microcline perthite. It is likely that the sandstone originated in an area of high relief or cold temperature because of the presence of feldspar in the sandstone [65].

Igneous, sedimentary, and metamorphic rock fragments are dominant in the sandstone. Most igneous rock fragments consist of volcanic clasts containing feldspar phenocrysts. There are three types of sedimentary rock fragments: sandstone, siltstone, and carbonates. In sandstone, lithic fragments indicate that the source area was located at high relief; these fragments originate from orogenic belts situated near suture belts and magmatic areas [66,67]. In addition to slate, schist, quartzite, and gneisses, mica schist are the most prominent metamorphic rock fragments. The studied samples reveal that low grade metamorphic rocks were exposed during the deposition of the Kamlial Formation in the source region. The Lower and Higher Himalayas of Kashmir are covered with slates, phyllites, and volcanic rocks. Sandstones containing moderate levels of muscovite and biotite indicate low grade metamorphic rock in the source area [68]. Mica flakes are generally bent and deformed (Fig. 6, Fig. 8F). It has been suggested that the bent mica originates from metamorphic rocks or deformed during diagenesis and tectonic activity [69]. Metamorphic, volcanic, and sedimentary rocks contributed to the Kamlial Formation.

Few thin sections contain accessory minerals such as tourmaline, zircon, epidote, hornblende, and chlorite (Fig. 6C, I, and K). Tourmaline has a greater concentration than epidote. Epidote and chlorite are heavy mineral assemblages that suggest metamorphic detritus [[70], [71], [72]]. It has been accepted that the presence of tourmaline and epidote indicates a low-to medium-grade metamorphic origin [73]. An acid rock source is supported by the presence of zircon (Fig. 6I) and rutile (Fig. 7H) [74]. It is possible that zircon originated from sedimentary and intermediate igneous rocks [75]. Granite and pegmatites may be the source of these sediments. A stable opaque form of zircon, rutile, and tourmaline are found in acidic igneous rocks or reworked sediments.

The recommended source lithologies for the considered sandstones are all located in northern Pakistan. On the provenance discrimination diagram of [56], the sandstone quartz, feldspar, and lithic data are plotted. The data falls under the category of recycled orogenic (Fig. 9). As discussed in previous sections, field observations and modal mineralogy data (Table 2, Table 3, Table 4) indicate that these rocks are associated with the Himalayan orogenic belt.

### 6.2 Depositional environment

During the Paleocene-Eocene collision of the Indian Plate with the Eurasian Plate, a large amount of detritus was found in the Southern Foreland Basin [10]. Kamlial Formation was deposited by sandy bed loads or by the load River. Sandy bed loads consist mainly of sand, but dispersed gravel is also present. A flood may result in a dense load of suspended sediment, which are temporarily deposited on the banks and channels of rivers. Fine sediments are rarely deposited due to their difficulty resisting erosion during flooding. A highly erodible bank results in a high width to depth ratio, as well as a high lateral moment across the whole channel zone within the area. So, there is a low degree of sinuosity and a reasonable degree of braiding. Sand availability is a major factor influencing braiding patterns [76].

Local slopes of high sinuosity or mixed loads flowing at right angles to the main river's flow direction deposited floodplain sand bodies interbedded with siltstone [13]. As a result of the limited amount of mudstone in Kamlial Formation, several factors may have been revealed, including (1) low subsiding rates that facilitated rapid lateral migration of channels and the regular erosion of flood basin deposits, (2) an arid climate regime and limited vegetation that allowed the channels to migrate laterally and to trap fine clastic particles more readily [76].

There is evidence that the Kamlial Formation was deposited continentally scale during the Miocene. Sedimentary structures could provide the most comprehensive evidence for understanding the environment in which they were deposited [77]. Conglomerates (Fig. 10E) indicate deposition in fluvial channels and shallow marine environments. Conglomerates are either intraformational or extra formational clasts. Extra formational clasts are primarily composed of volcanic clasts. The load casts (Fig. 10D) are formed on the underside of denser layers such as sand that are superimposed on denser layers such as silt and shale. The load casts display different depositional environments, such as fluvial and shallow-marine environments. Ripple marks (Fig. 10I) in the study area suggest that the tidal flat environment affects the deposition [78]. The petrified wood (Fig. 10B) indicates the continental origin. Additionally, it shows that a portion of wood has been completely transformed into rock where silica replaced all organic material. However, wood retains its original shape.

### 6.3 Diagenesis of Kamlial Formation

During the deposition and uplifting of sedimentary rocks, all changes in the texture composition and other physical characteristics present in the sediments are called diagenesis [[79], [80], [81], [82]]. Kamlial sandstone petrography exhibits diagenesis changes such as compaction, pressure solution, silica cementation, calcite cementation, and ferruginous cementation. Further details of each section are described below.

#### 6.3.1 Compaction

Compression normally occurs due to vertical shear-compressional stresses due to the increasing weight of overburden, as well as under compressional tectonic forces. During compaction, quartz grains were fractured and mica grains were bent. In addition, quartz grains exhibit a variety of contacts, including point contacts, and long contacts, as well as concave-convex contacts. Deformation and bending of mica (Fig. 8F), stretching and fractures of quartz (Fig. 6D), and metamorphic rock fragments (Fig. 8B–E) are evident of epi-diagenetic processes and provide evidence of tectonic uplift in the area. There is evidence of highly plastic deformation of muscovite in the studied thin sections.

#### 6.3.2 Pressure solution

Stylolites are formed when cemented rocks are subjected to overload and tectonic pressure [83]. Pressure solution forms sutured contacts between quartz grains [84]. In the present study pressure solution is observed in thin sections (Fig. 6L).

#### 6.3.3 Cementation

Ferruginous cement and calcite are generally found as cementing materials in sandstone (Figs. 6F, 7B,F and 8C). There are calcite and ferruginous cements surrounding the sandstone grains. The presence of calcite veins indicates a late diagenesis event. Calcite replaces quartz in allowing a better understanding of how diagenesis takes place in water due to changes in pH (hydrogen ions) and Eh (redox potential). Carbonates differ from silicates in that carbonate reactions occur faster, especially at low temperatures. At shallow depths, this can result in carbonate dissolution and cementation [85]. Ferruginous cement is found in an oxidizing environment. Minerals such as hematite indicate precipitation in an oxidizing environment [[86], [87], [88], [89]]. As hematite coats grains, it forms a thin layer. Additionally, it develops in the cleavage plane. Leaching of iron occurs at the cleavage plane of biotite. Cementing materials were also produced by iron release from minerals during diagenetic processes. As a result of iron cement, sandstone turned reddish to brownish. Sandstone formed silica cementation due to the chemical weathering of feldspar. During diagenesis, quartz dissolves and is replaced by calcite, which contains silica.

#### 6.3.4 Authigenic clay minerals

Diagenesis is responsible for the formation of Authigenic clay minerals in the Kamlial Formation sandstone. Digenesis is strongly influenced by mineralogy and depositional environment. Precipitation of formation water can lead to the formation of authigenic clay minerals in sandstones [90]. In addition to these changes, autigenesis may occur when feldspar and volcanic glass are transformed into clay minerals or when one clay mineral type is transformed into another. Clay minerals were formed during feldspar replacement. Replacement occurred along fractures and cleavages. Feldspar grains often partially or completely replaced by sericite.

## 7 Conclusion

1) The Kamlial Formation contains quartz grains that are angular to sub-angular, suggesting that they were sourced locally, whereas grains that are rounded to sub-rounded were transported far and became rounded.
2) Microscopic analysis classified the Kamlial Formation sandstone as lithic arenite.
3) In the Kamlial Formation, feldspar, plagioclase, and perthite indicate they were derived from granites and granite gneisses. The low feldspar content of lithic arenite indicates semi-humid conditions during deposition. A granite source would be acidic plutonic igneous rock with monocrystalline quartz grains. Quartz grains with undulations and polycrystalline grains indicate metamorphic sources including slate, schist, and gneiss. Several accessory minerals are found in the Kamlial Formation, suggesting granites, schists, and granite gneisses were their source lithologies.
4) Using the triangular diagram plotted for the provenance analysis, lithic arenites are placed in the field of recycled orogeny.
5) The Kamlial Formations in the foreland basin were deposited with calcite veins. It was caused by changing conditions from acidic to alkaline. Kamlial Formation was deposited in a fluvial environment, according to field and petrographic data. Pakistan's north region provided most sedimentary materials for the Kamlial Formation. Slates, biotite schist, quartzite, and volcanic clasts are among the interformational clasts in the Kamlial Formation. Sedimentary materials were also deposited in the Himalayan foreland basin from adjacent areas after the continental collision between the Indian and Eurasian Plates. All diagenesis changes observed in petrography are examples of compression, pressure solution, and cementation.

## Funding

This project is funded by 10.13039/501100002383King Saud University, Riyadh, Saudi Arabia.

## Data availability

Data will be made available on request.

## Additional information

No additional information is available for this paper.

## CRediT authorship contribution statement

**Musa Khan:** Writing – review & editing, Writing – original draft, Visualization, Validation, Software, Methodology, Investigation, Formal analysis, Data curation, Conceptualization. **Rehan Khan:** Validation, Supervision, Methodology, Investigation, Formal analysis, Data curation. **Shams Ul Islam:** Visualization, Supervision, Investigation. **Asad Khan:** Validation, Supervision. **Yanmei Zhong:** Visualization, Resources. **Fuad A. Awwad:** Writing – review & editing, Resources. **Emad A.A. Ismail:** Writing – review & editing, Resources.

## Declaration of competing interest

The authors declare that they have no known competing financial interests or personal relationships that could have appeared to influence the work reported in this paper.
